# A Practical Approach to the Diagnosis of Lyme Borreliosis: From Clinical Heterogeneity to Laboratory Methods

**DOI:** 10.3389/fmed.2020.00265

**Published:** 2020-07-23

**Authors:** Giusto Trevisan, Serena Bonin, Maurizio Ruscio

**Affiliations:** ^1^DSM-Department of Medical Sciences, University of Trieste, Trieste, Italy; ^2^ASU GI-Azienda Sanitaria Universitaria Giuliano Isontina, Trieste, Italy

**Keywords:** Lyme disease, Borrelia, clinical symptoms, diagnosis, clinical heterogeneity

## Abstract

Clinical evaluation of Lyme Borreliosis (LB) is the starting point for its diagnosis. The patient's medical history and clinical symptoms are fundamental for disease recognition. The heterogeneity in clinical manifestations of LB can be related to different causes, including the different strains of Borrelia, possible co-infection with other tick transmitted pathogens, and its interactions with the human host. This review aims at describing the heterogeneous symptoms of Lyme Borreliosis, as well as offering a practical approach for recognition of the disease, both in terms of clinical features and diagnostic/research tools.

## Introduction

The genus Borrelia includes three Groups: Lyme Borreliosis (LB), Reptil Associated (REP), and Relapsing Fever (RF) Group (1).

Lyme disease or Lyme borreliosis (LB) is an anthropozoonosis, caused by different genospecies of the *Borrelia burgdorferi sensu lato* complex. The main tick vector for Borrelia species in Europe is the *Ixodes ricinus* (2), in America the *Ixodes scapularis* and *Ixodes pacificus* (3–5), while in Asia (6) and Russia (7) it is the *Ixodes persulcatus*. These ticks are possible vectors of Lyme Borreliosis (LB) as well as other pathogens, including viruses, intracellular bacteria, and Protozoa which can co-infect humans (LB co-infections) (8, 9). There are several *B. burgdorferi sensu lato* genospecies, directly associated with human LB. However, only three genospecies, namely *Borrelia burgdorferi sensu stricto, B. afzelii*, and *B. garinii*, have been systemically related to LB (4, 10). In addition, four other genospecies have been occasionally detected in humans: *B. bissettiae* (4, 5), *B. lusitaniae* (6, 7), *B. spielmanii* (8), and *B. valaisiana* (9), especially in Europe (11). Specificity in terms of dominating hosts has been reported both across and within continents (12, 13). The spatial distribution of the different genospecies allocates *Borrelia burgdorferi sensu stricto* in North America [and possibly *B. mayonii*, although this causes a disease somewhat distinct from typical LB (14)] and five species in Europe and Asia, *B. afzelii, B. garinii, B. burgdorferi, B. spielmanii*, and *B. bavariensis* (15). The heterogeneity in terms of genospecies can mirror different clinical manifestations of LB due to host specialization and tissue tropism. Although overlapping, distinct spectra of clinical manifestations have been recognized for the three main genospecies. In detail, *B. burgdorferi sensu stricto* is mostly associated with arthritis and neuroborreliosis, *B. garinii* with neuroborreliosis, and *B. afzelii* with chronic skin conditions such as acrodermatitis chronica atrophicans (10).

Spirochetes circulate in small amounts in the blood even in acute LB patients (16), with the exception of *Borrelia mayonii* which has been reported to cause high spirochetemia (14, 17). Depending on the case and genospecies, they can grow in several tissues (18), including skin, nervous and joint system, although less frequently LB can also affect eyes, heart, spleen, and other tissues.

Based on the spatial variability of Borrelia, for an accurate diagnosis, it could be useful to know if the patient has visited other countries or continents.

**Figure 1:**
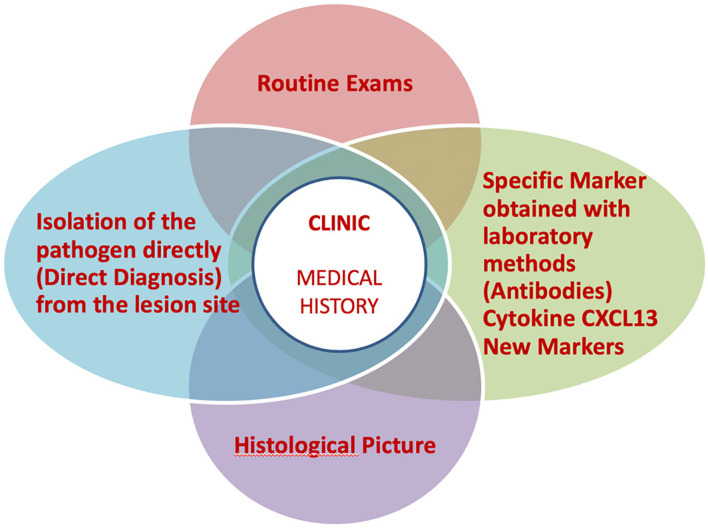
Overview of LB management.

Some clinical aspects that can be helpful for a correct diagnosis of LB will be described hereafter. Figure 1, instead, shows an overview of possible overlapping scenarios defining LB. Furthermore, a brief description of laboratory investigation tools is included at the end of the review.

## Tick-Bite Without Erythema Migrans

Patients sometimes seek medical assistance after a tick bite. In this case, the first step is to remove the tick with small tweezers or an *ad hoc* tool at the level of the rostrum. Afterwards, it is important to inform the patient of the symptoms, which, in the case of Borrelia infection, may develop in days/weeks. It is also possible to submit the tick for identification and testing for different pathogens. The identification of pathogens within the tick defines a possibility, not the certainty of developing LB (19).

## Erythema Migrans (EM)

**Figure 2:**
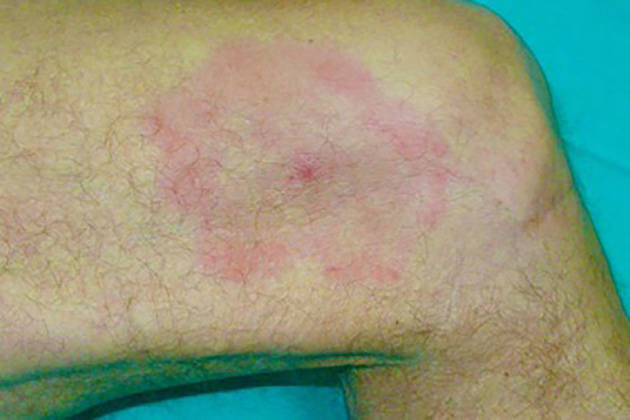
Erythema Migrans of the thigh.

Recognition of an EM rash is very important in LB as it is a hallmark symptom of LB, even when the patient does not recall the tick bite. However, as it has been observed, in rare cases the tick can still be attached to the center of the EM (20, 21). The geographical area where the patient was bitten as well as the date are important elements that should be gathered from the patient. Other variables to establish are: the time elapsed between the tick bite and the appearance of the erythema (usually 5–30 days) and its diameter, especially if larger than 5 cm (22). The most important diagnostic criterion is the EM centrifugal evolution. Erythema migrans (Figure 2) is pathognomonic for LB, therefore it should be treated immediately as serology testing to confirm infection is not necessary. Nevertheless, the clinical presentation of an EM can vary considerably (23). Several clinical variations have been observed, such as smaller-sized-EM of about the size of a coin, oval shaped EM with no darker outline, red-violet EM (erysipeloid), EM with vesicles which mimics herpes simplex or herpes zoster (24), painful EM (burning), itchy EM, hidden EM (scalp), and EM with atrophic evolution (25). It has been shown that in some cases of EM, Borrelia infection can already be disseminated (26).

Differential diagnoses include: mycosis fungoides, granuloma annulare, and interstitial granulomatous dermatitis (IGD), tinea corporis (mini EM), and erythema necroticans migrans.

Serological testing is not recommended because of their poor sensitivity in the early stages of LB. In order to achieve the best outcome for patients, antibiotic treatment should be started without delay.

## Cutaneous Manifestations Excluding the Erythema Migrans

### Multiple Annular Erythema

Secondary EM is characterized by multiple erythematous lesions, which do not develop round the site of the tick bite. It can consist of a few or several plaques that can be located throughout the body (27). The lesions are multiple and can vary from a few cm to more than 20 cm, and are more frequently observed in children (22). The presence of multiple annular erythemas may precede the onset of neurological manifestations, especially in adults.

### Borrelia Lymphocytoma

**Figure 3:**
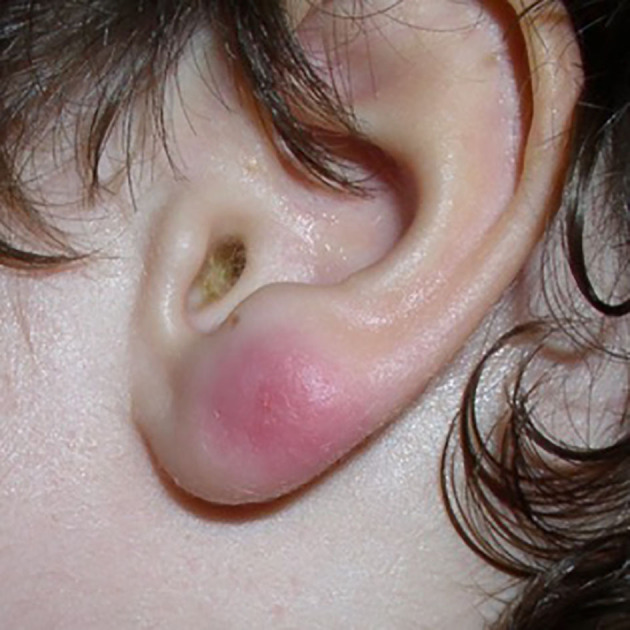
Borrelia Lymphocytoma of the ear lobe.

Borrelia lymphocytoma is defined as a B-cell pseudo-lymphoma that occurs in response to the presence of Borrelia antigens in the skin. Borrelial lymphocytoma can develop when EM is present and mimics a tick-bite reactive nodule. It is relatively frequent in Europe, while it is seldom observed in the US, because in most cases it is caused by *Borrelia afzelii* and more rarely by *B. garinii* and *B*. *bissettii* (28). Clinically, it appears as a solitary (rarely multiple) soft and non-tender bluish-red nodule or plaque with a size between 1 and 5 cm, sharply demarcated. It is typically found on the ear lobe (Figure 3), the mammary areola, and less frequently on the scrotum or the axillary fold. Extra-cutaneous signs and symptoms are very infrequent. The presence of Borrelia biofilm in human infected skin tissues has been demonstrated (29).

In the presence of this clinical manifestation the following exams should be performed: serology for *Borrelia burgdorferi* (ELISA and Western-Blot), **β**2-Microglobulin, and serological tests for Ehrlichia (Anaplasma) (30). Histological examination of skin biopsy and immunohistochemistry to define immunophenotype are also suggested (usually CD20 positive, Bcl-2 negative, κ and λ light chain expressed in an equivalent manner and Borrelia-PCR on DNA from skin slides).

**Figure 4:**
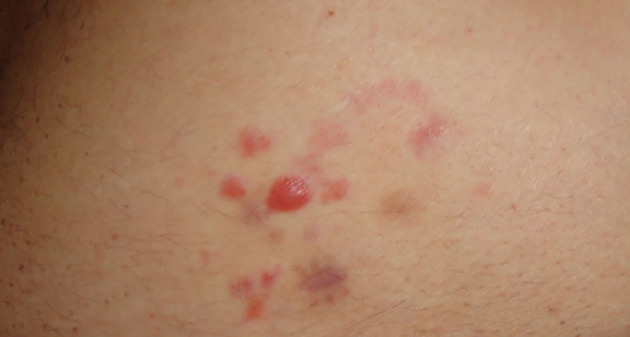
Primary cutaneous B cell marginal zone lymphoma of the trunk. Of note the image that has been already published refers to the same patient but it is slightly different from this one.

Differential diagnosis includes cutaneous marginal zone lymphoma (PCMZL, Figure 4), which clinically and histologically may present similarities at the immunophenotype. PCMZL is generally CD20, CD22, CD79a, and BCL-2 positive, whereas it is CD5, CD10, Bcl-6, and CD23 negative, and the κ/λ light chain ratio in the histological tissue is very high (31). Borrelia's detection in PCMZL is included in the EORTC guidelines (32, 33).

PCR for Borrelia on tissue's DNA (frozen or formalin-fixed and paraffin-embedded) can target OspA as reported by Cerroni (34), but also p41 (flagellin) and p66 (35). Skin biopsy specimens from the site of the lesion can also be submitted for culture and isolation of Borrelia.

### Acrodermatitis Chronica Atrophicans (ACA)

ACA is the pathognomonic symptom of late LB. Patients, at presentation, should be asked whether they remember being bitten by a tick several months or even years before and whether they ever had an EM. Since the clinical appearance of ACA is not distinctive, it is of key importance to be generally alerted of the possibility of ACA in patients with bluish-red discoloration of a limb with or without swelling and/or atrophy, especially where LB is endemic (36, 37).

**Figure 5:**
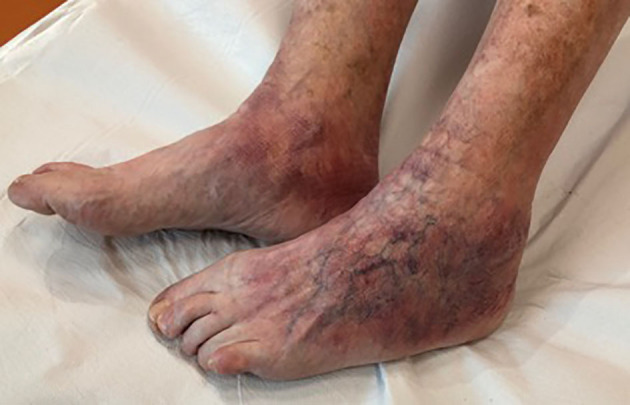
Acrodermatitis chronica atroficans of the legs.

Unilateral acrocyanosis is present in the initial phases. This feature is followed by atrophy of the upper and/or lower limbs in an asymmetric manner, which, due to thinning and consequent greater transparency of the skin, allows the vessels of the dermis to be more visible. This condition leads over time to thinning of the most involved limb (22). ACA (Figure 5) is usually localized on the limbs, however, the face is also an acral site, and in some cases, it is difficult to distinguish the ACA of the face from Parry-Romberg syndrome, which may be a variant (38).

In addition to ACA, in some cases, other atrophic-sclerodermic manifestations may be related to LB (39, 40).

Serology by chemiluminescence is usually very high in VlsE IgG; in Western-Blot, p93 (p83/100) and DbpA are generally observed.

Skin biopsy for histological examination and PCR for Borrelia are also possible for research purposes. Isolation of Borrelia in BSK medium from skin lesion can result in the growth of *Borrelia afzelii* (or more rarely *valaisania, lusitaniae*, or *yangtze*).

### Other Possible Skin Manifestations

Other possible skin manifestations that have been associated with LB are: urticaria (41), purpura (42), and erythema nodosum (Baggio-Yoshinari syndrome) (43).

## General and Extracutaneous Clinical Manifestations

### General Symptoms

Important information to be obtained from patients includes: the geographical area where the patient lives (if endemic or not for LB), if, in the previous weeks or months the patient has been in wooded areas, if he/she has traveled or has been camping, or has spent time in public parks and gardens or if he/she owns any pets. Requested information should also include the date of the onset of symptoms, recollection of a tick bite and/or of a circular erythema as well as the location and the duration of the skin lesion. In the case of a positive, response, the patient should be asked if he/she was previously treated with antibiotics, what type of antibiotics, and what the duration of treatment was. Other clinical manifestations can be fever, lymphadenopathy, balance disorders, dizziness, and photophobia (44).

### Joint and/or Muscular Symptoms

Arthritis occurs after 4 days to 2 years (average, 6 months) from EM (45–49). In a European group of patients, the period between the tick bite or EM to the onset of arthritis ranged from 10 days to 16 months, with an average of 3 months (50). A summary of the articular involvement of LB is reported in Table 1.

**Table 1:** Articular Involvement in LB.

**Features**	**Location**	**Clinic**
Mono/Oligo Involvement	Large Joints	Swelling
Asymmetry frequent attacks	Knee Joint most affected	Marked functional Impotence
		Skin Nodules
		Absence of Stiffness in the Morning

In the early phase, the patient presents mono- or oligoarticular migrant arthralgia at the level of the large joints. The first affected joint is often near the site of the EM or the tick bite. However, sometimes other large or small joints, such as the temporomandibular joint (TMA), are also affected (51). Over time, the duration of joint arthralgia tends to lengthen, while painless intervals become shorter.

The articular involvement in the late phase has different clinical features compared to the typical migrant myo-arthralgia of early LB. The clinical symptomatology is not easy to distinguish from arthritis due to other causes. The disorder can become chronic or intermittent, with attacks lasting from a couple of weeks to a few months, which can be followed by resolution of symptoms. The intensity of the attacks decreases over time. Hyperpyrexia is not usually present, but a general sense of fatigue is common.

Swelling of the joints with marked functional impotence is often present. Affected knees, for instance, may have very large effusions (synovial fluid) (52). If those injuries are not diagnosed and treated, the patient will possibly experience erosion of the cartilage and bone which can lead to permanent damage of the joint.

Muscular system involvement includes myalgia, muscle weakness, and myositis (53) with difficulty in raising the arms above the head, carrying weights, and climbing stairs; and dysphagia, with difficulty breathing due to the involvement of intercostal muscles (inter-costal diaphragm). In some cases, these symptoms can simulate a dermatomyositis (41).

To confirm diagnosis, it is useful to perform a serological ELISA test followed by a Western Blot. In case the patient reports having headaches and/or a fever, tests for TBE, Ehrlichia (Anaplasma), Rickettsia, and Bartonella coinfections are suggested. A rheumatologic examination can be also requested.

Serum IgG antibodies for *B. burgdorferi s.l*. are present in high titers in patients with Lyme arthritis, while a negative IgG serology rules out the diagnosis (54, 55). Serological investigation of synovial fluid is not helpful because of the absence of a blood–synovial barrier; IgG antibody concentration in serum and synovial fluid will be equivalent.

In some cases, it can be useful to perform a PCR for Borrelia using DNA from synovial fluid or from a biopsy fragment of the synovium (56).

If the clinical picture is suggestive of LB, but the serology is negative, the clinical symptoms should over-rule a negative test, as pointed out by Burgdorfer. Commercial test kits are often inaccurate and can give negative results even in advanced LB. A negative test does not demonstrate the absence of LB and further investigations are needed to rule out differential diagnoses, such as that for an autoimmune disease (57).

### Neurological Symptoms

Involvement of the nervous system occurs in up to 15% of patients with untreated LB (58). A summary of the possible neurological manifestations in LB is reported in Table 2.

**Table 2:** Neurological Involvement in LB.

**Lymphocytic Meningitis**	
**Cranial neuritis**	Facial palsy
	Cranial nerves palsies of III, IV, VI
	Optic neuritis and optic atrophy
**Meningoradiculitis**	Garin-Bujadoux-Bannwarth syndrome
**Myelitis**	Monofocal lesion
	Multifocal lesions
	Acute transverse Myelitis
**Encephalitis**	Loss of consciousness
	Speech disorders
	Recent cognitive disorders
	Affective disorders
**Cerebral vasculitis**	
**Pseudo tumor cerebri**	
**Peripheral neuropathy**	**Chronic asymmetric neuropathy**
	Small fiber neuropathy
**Psychiatric disorders**	States of anxiety
	Depression
	States of panic

Headache is the most frequent symptom. Cranial nerve involvement may occur, particularly that of the facial nerve (80%). Facial paralysis is bilateral in 25% (59, 60). Paralysis of the III, IV, VI cranial nerve, and optic neuritis can be observed.

Among children in Europe, the most common manifestations are facial nerve palsy (about 55%) and lymphocytic meningitis (about 30%) (61).

Meningopolyneuritis (Garin-Bujadoux-Bannwarth) with radicular pain and sometimes paresis of extremities or the abdominal wall (62, 63), neurologic bladder (64), and paresthesia can be observed. Myelitis is a rare manifestation of LB; although monofocal or multifocal lesions of the cervical spinal cord (65) have been described, as well as lombosacral myelitis (66) and acute transverse myelitis.

Pseudo tumor cerebri associated with LB was first described in 1985 (67). Subsequently, other cases have been described mainly in children (68) and rarely in adults (69).

Infection of the central nervous system is observed in 2–4% of Lyme neuroborreliosis, typically in the late or chronic stage of the disease (70). Encephalitis presents non-specific MRI findings of diffuse involvement of the brain parenchyma. Cerebral, cerebellar parenchyma, and thalami can be involved (71).

Neuroborreliosis can be associated with speech disorders, recent cognitive, and affective disorders (72), psychiatric disorders, states of anxiety, depression (73), and states of panic, and restless syndrome can be related to LB (74).

Cerebral vasculitis in patients with LB is observed in about 0.3% of cases (75). In some cases, the possibility of infection or co-infection (76) with *Borrelia miyamotoi*, which can be transmitted by the same tick as LB, should be considered (77, 78).

Neurological examination is suggested in order to rule out a differential diagnosis. In addition to the serological tests for anti-Borrelia antibodies by ELISA and Western Blot, it is also possible to perform a PCR for the detection of Borrelia DNA in cerebrospinal fluid (79) as well as an ELISA for Chemokine 13 (80).

Peripheral neuropathy can be detected in about 5–10% of Lyme neuroborreliosis cases. It can present as a chronic asymmetric neuropathy, usually without intrathecal antibodies (81).

For late neuroborreliosis, a careful examination is suggested for possible acrodermatitis chronica atrophicans (acral acrocyanotic appearance, and to verify any differences in limbs diameter) (82), and possibly a biopsy (for example on the ankle presenting neuropathic alterations) for histological examination of the small nervous fibers. Small fiber neuropathy (SFN) can be observed after antibiotic treatment (Post-treatment Lyme disease syndrome—PTLDS) and may be responsible for sensory symptoms (83).

In most patients, examination of the cerebrospinal fluid (CSF) reveals lymphocytic pleocytosis, damage to the blood-CSF-barrier, and an intrathecal synthesis of immunoglobulin IgM, IgG, and sometimes IgA (84); the protidorrachia is normal or slightly increased; the glycorrachia is normal or only slightly diminished.

During paralysis of the facial nerve, the CSF often presents lymphocytic pleocytosis even in the absence of signs and symptoms of meningitis (85).

After the onset of neurological symptoms, for a short time, intrathecal synthesis may not be detectable and CSF pleocytosis may be absent especially in children with isolated paralysis of the seventh cranial nerve (86). The production of intrathecal antibodies can continue even after recovery. On the other hand, intrathecal synthesis of specific antibodies is lacking in many patients with neuroborreliosis.

The use of chemokine (C–X–C motif) ligand 13 (CXCL13), a B-cell attracting chemokine, was debated for the laboratory diagnosis of acute Lyme neuroborreliosis in CSF (87). CXCL13 can be detected in CSF early in the disease and it has been reported to decrease with treatment (88). However, CXCL13 is not specific for Lyme neuroborreliosis and can also be found in some other inflammatory diseases of the CNS (88).

The different genospecies are often related to different clinical manifestations. *Borrelia garinii* is mainly related to typical early Lyme Neuroborreliosis (i.e., pain, meningoradiculoneuritis, or Bannwarth syndrome) while *Borrelia valaisiana* causes neurologic Lyme manifestations less frequently (89); *Borrelia afzelii* is less specific for neurologic manifestations as radicular pain and meningeal symptoms are rarely present (79). It is observed more often in late Neuroborreliosis by diffusion from the skin to small nerve fibers, often deriving from Acrodermatitis chronica atrophicans (82). It is able to cross the blood-brain barrier, but has a limited ability to produce inflammation in the CSF. The role of this genospecies has yet to be fully clarified.

### Heart Symptoms

The involvement of the heart is observed in 4–10% of patients with LB, of whom 90% have Lyme carditis (90, 91). The most frequent manifestations are:

- Atrioventricular Conduction disorder or other rhythm disorders,
- Myocarditis (92, 93),
- Pericarditis (94),
- Postural Orthostatic Tachycardia Syndrome (POTS) (95).

In addition to dyspnea, chest pain, or irregular heartbeat, typical symptoms include syncope episodes (93). On physical examination, 35% of patients had bradycardia and about 15% tachycardia.

If heart involvement in LB is suspected, a cardiological examination is suggested. The following investigations should be addressed: 12-channel ECG and 24-h ECG Holter (query: rhythm analysis, PQ interval, QRS width, ectopic beats), chest X-ray (question: heart size, congestion); echocardiography (diameter, ejection fraction, abnormal wall movement, pericardial effusion); cardiac MRI, and in selected cases myocardial biopsy for histological examination and cultural isolation of Borrelia (96). Electrophysiological examination can be done only in selected cases to confirm the diagnosis and establish a prognosis, as it is a highly invasive procedure and can cause arrhythmia. Patients should be clearly informed about the procedure and its associated risk.

### Ocular Symptoms

Ocular manifestations can be linked to a direct involvement of the eye or can be secondary to Neuroborreliosis. Ocular involvement, is possible at every stage of LB and they can be summarized as follows:

- Follicular conjunctivitis often self-limited, and,
- Photophobia.

They can appear in the first stages of LB.

In the early disseminated phase, these manifestations are possible:

- Macular edema,
- Uveitis and Iridocyclitis,
- Optic Neuritis and Neuroretinitis,
- Retinal Vasculitis and Choroiditis,
- Branch Retinal Vein Occlusion (BRVO) (97),
- White Dot Syndrome (98),
- Stromal Keratitis and Episcleritis.

Intermediate uveitis is the most common uveitis in LB. Posterior uveitis is mostly associated with chorioretinal involvement (99).

Keratitis is characteristic of the second and third stages of LB and may either be interstitial or ulcerative. Episcleritis and scleritis are rare and can be observed mainly in the late phase of LB (100).

Regarding ocular manifestations due to Neuroborreliosis, they include:

- Myositis of Extraocular Muscles,
- Facial Palsy and other Cranial Nerve Palsies (101),
- Horner's syndrome (102).

## When to Suspect Coinfections

Coinfections should be suspected in the following cases (103, 104):

1. ✓ in the presence of fever and headache,
2. ✓ in patients diagnosed with LB, who do not clinically improve or,
3. ✓ whose symptoms have changed (e.g., appearance of febrile episodes) after adequate antibiotic treatment,
4. ✓ when patients have leukopenia and neutropenia, persistent after treatment, or high ESR,
5. ✓ when patients present purple, persistent skin lesions, even the same purpuric Erythema migrans (in our experience).

In these cases tests for Rickettsia, Anaplasma (105), Bartonella, Babesia (106), and TBE (FSME Frühsommer-Meningoenzephalitis) (105, 107) and Powassan virus (108) are suggested.

## Occasional Positivity of Anti-Borrelia Antibodies

The spirochetes may persist in affected organs even months to years after the initial infection, causing a chronic form of illness. Therefore, antimicrobial agents have been found to have a role in all stages of the disease (109).

When patients come to the Lyme Disease Center, because they have been found to be positive for anti-Borrelia antibodies, it is necessary to request an accurate medical history including the geographical area where the patient lives, recollection of a tick bite, and if applicable, the recollection of a circular rash, its possible location, and its duration. This collection of information should be followed by an accurate examination for the presence of LB related symptoms. Medical history should also include any previous antibiotic treatment.

In the absence of any reported tick bite or EM and related clinical manifestations, if the serological test results are positive in IgG antibodies it is recommended to perform a WB, whereas positive IgM may not be specific, and serology should be repeated after 6 months.

When the skin, the myo-articular system, and/or the nervous, cardiac or ocular systems are involved, specific investigations must be carried out, as indicated in the two previous paragraphs.

These patients should also be subjected to immunological testing, as Borrelia antigens can induce autoimmune diseases in predisposed subjects (Trigger Factor).

In some cases, Borrelia induces the production of antibodies against certain surface antigens, which cross-react with specific sequences of organism structures (antigenic camouflage). It is known, in fact, that there can be cross-reactivity between OspA and the human leukocyte function antigen (LFA) (110, 111), as well as between Osp and acetylcholine receptors, enolase gamma, and Borrelia Enolase (112).

A thorough diagnostic examination should be based on the clinical picture, the organs involved, the serological pattern, and the tests that have been already performed.

## Persistence of the Clinical Manifestations after Treatment

The persistence of symptoms related to LB can be observed in untreated patients as well as in patients who have undergone treatment but continue to present symptoms. Untreated patients can develop persistent signs and symptoms, which usually involve the joints and less commonly the nervous system (113). Patients who instead have been treated mainly report a worsening of subjective symptoms. After 6 months, 36% of patients experienced an increase in fatigue, 20% complained of widespread pain, and 45% of neurocognitive impairment (114). Long-term persistent illness following antibiotic treatment is not uncommon, especially when treatment is delayed. About 10–20% of patients treated for early or late LB experience persistent symptoms, which may last for months or years (115). Symptoms consist of fatigue, joint and muscle pains, recent cognitive disorders, root pain, paresthesia, or dysesthesia. If we analyze the group of patients treated for Neuroborreliosis, this percentage increases significantly. Eikeland found that in Europe only 56% of patients treated with antibiotics for neuroborreliosis were symptom-free 30 months after treatment (116, 117).

Some published authors of medical research recognize mainly two clinical scenarios: the first characterized by typical symptoms of post-Lyme disease when symptoms persist for <6 months, and post-treatment Lyme disease syndrome or chronic Lyme disease if symptoms are debilitating and persist after treatment (118).

In the International Lyme and associated diseases society (ILADS) guidelines, “chronic Lyme disease” is described as a multisystem illness with persistent symptoms (119, 120), including fatigue, cognitive dysfunction, headaches, sleep disturbances, and other neurologic features, such as demyelinating disease, peripheral neuropathy, and sometimes motor neuron disease, neuropsychiatric presentations, cardiac presentations (including electrical conduction delays and dilated cardiomyopathy), and musculoskeletal problems (121–123). The cause may consist in residual damage to tissues and the immune system and cytokine production (122, 123), which occurs as a consequence of the infection causing possible modification of protein antigens located on the cell membrane. According to certain controlled studies, post-treatment Lyme disease syndrome (PTLDS) has often been shown to be non-responsive to antibiotic therapy. Several hypotheses have been suggested in order to explain PTLDS, among them, the presence of bacterial debris, autoimmunity, and co-infections, (120, 124, 125). In several studies, persistent Borrelia was isolated by culture or PCR (126–139).

The effectiveness of Ceftriaxone in several cases supports the hypothesis of bacterial persisters which survive in spite of previous antibiotic treatment (140). Delong et al. (140) have reported that retreatment can be effective, but further studies are needed to assess the role of antibiotics for persistent infection. It has been demonstrated that the persistence of *Borrelia burgdorferi* is likely due to the development of biologically less active permanent forms (Spheroblasts and round shapes) and of biofilm (141, 142). Biofilm analysis (Clinical Biofilm Ring Test—cBRT) (143) and treatment can produce an improvement in test results (144). In some cases, Borrelia can induce the production of antibodies against certain surface antigens, which cross-react with specific sequences of organism structures (antigenic camouflage). OspA is known, in fact, to cross-react with LFA, as well as Osp with Acetylcholine receptors. Treatment of *B. burgdorferi* in the stationary phase can result in a higher probability of regrowth once antibiotic treatment is interrupted (119).

Post-Treatment Lyme Disease Symptoms (PTLDS) and Chronic Fatigue Syndrome/Myalgic Encephalomyelitis (CFS/ME) have several clinical features in common, including fatigue, musculoskeletal pain, and cognitive difficulties. The Canadian Clinical Criteria for CFS/ME diagnosis include the following symptoms: Fatigue > 6 months, limited physical activity, unrefreshing sleep, impaired thinking and speech, vertigo, post-exertional fatigue, stress induced by exertion, reduced concentration, orthostatic intolerance, food intolerance (145).

Immunologic mechanisms have been suspected to play a role in both PTLDS and CFS/ME.

In CFS/ME patients, serum Activin B levels were significantly elevated compared with control subjects. Elevated Activin B levels together with normal Activin A levels identified patients with the diagnostic symptoms of CFS/ME (146, 147).

It has also been hypothesized that there is an immunosignature specific to CFS/ME and that this could aid the diagnosis. Scientists were in fact able to identify a 256-peptide signature that separates CFS/ME samples from healthy controls (148).

An increase in levels and frequency of IgG anti-neural antibody reactivity has been found in PTLDS. The anti-neural antibody response was independent from serologic positivity for antibodies to *Borrelia burgdorferi*; however there was no significant difference in the prevalence of anti-neural antibody reactivity between CFS/ME patients and healthy controls (149).

## Pregnancy and Pediatric Case Assessment

It is documented that trans-placental transmission of the spirochetes from the mother to the fetus is possible, and Borrelia starts crossing the placenta (150, 151) during the first month, unlike Treponema, which passes through the placenta barrier starting from the 5th month. A case of congenital Lyme with multiple annular erythema at birth has been reported in a child whose mother reported having an erythema migrans during pregnancy. Culture of skin biopsy from the child‘s skin lesion was positive for *Borrelia garinii* and rapid recovery was achieved after antibiotic therapy (152). A study on seven pregnant European women with EM and Borrelia isolated from blood indicated that the course and outcome of early LB was uneventful when pregnant women were treated with intravenous ceftriaxone, and that the outcome of their pregnancies was good (153). Therefore, in case of pregnancy, antibiotic prophylaxis treatment may be appropriate in the case of tick bites in endemic areas.

Below is a description of the symptoms of LB in children with potential exposure to tick bites, who have been diagnosed with EM or positive serological results or clinical manifestations compatible with LB.

Clinical suspicion of Lyme disease is based on the following clinical manifestations: for early localized LB, the presence of erythema migrans, often on the face, possibly associated with conjunctivitis and/or photophobia; for early disseminated LB the presence of multiple annular erythemas, Borrelial lymphocytoma, cranial neuritis, headache and/or pain and stiffness in the neck, migrant myo-arthralgia with possible involvement of the temporomandibular joint, alterations of electrocardiogram suggestive of carditis; for late BL the presence of arthritis. Acrodermatitis chronica atrophicans can also occur in children, but it is rare (154).

Patients with non-specific symptoms (e.g., fever or fatigue without specific manifestations of early, disseminated or late Lyme disease) are classified as probably not affected by Lyme disease. These patients should be considered positive only if, after 1 month, serology tests demonstrate serum conversion.

In some cases a rapid test response is required, ELISA or CLIA (155). Clinical evaluation plays a fundamental role when having to make initial decisions regarding children who visit the pediatric emergency room.

## Detection of Borrelia in Clinical Samples

### Indirect Methods of Borrelia Detection

#### Detection of Antibodies Against *Borrelia burgdorferi sensu lato* Complex

Several commercial products are available for detecting IgG and/or IgM antibodies against *Borrelia burgdorferi s.l*. complex. Test systems comprise different techniques including the Enzyme-linked immunosorbent assay (ELISA), the Enzyme-Immunoassay (EIA), the Enzyme-Linked Fluorescence Assay (ELFA), the Chemoluminescence Immunoassay (CLIA), Luminex, Fluoro-Immunoassay (FIA), and Western Blots/Immunoblots. Some tests use antigens obtained from native Borrelia bacteria, whilst others use manufacturing methods to prepare recombinant antigens. In some assays a mixture of both are used.

The European and North American guidelines indicate that the diagnosis of LB is currently based on a two-tier serology at all stages of the infection, except when erythema migrans is present (156). The two-tier testing procedure includes ELISA or EIA or VlsE/C6 as the first test and a Western Blot/Immunoblot assay as a confirmatory test. The VlsE Complex (variable major protein-like sequence Expressed—Vmp 35 kDa) is a surface protein formed by three defined domains: two invariable constant regions at the COOH and NH_2_ terminals, and one internal variable region. The invariable, internal areas are masked and protected by the “*in vivo*” external variable regions. Due to the continuous modifications of its external antigenically variable component, Borrelia is able to escape the immune system.

After the death of the spirochetes, the VlsE protein is presented in its entirety to the immune system, which can thus induce the production of antibodies against the preserved and invariable regions of VlsE. The dosage of the VlsE protein and its sixth invariant region (IR6) peptide of *Borrelia burgdorferi* has been reported to quantitatively vary after antibiotic treatment (157–159), although VlsE and C6 are detected both in convalescent and healthy people, and thus they do not differentiate between active and past infection. OspC is used for detection of specific IgM antibodies in the first stage of the serologic test, either as a single antigen or as a mixture with other antigens.

Immunoblot (western blot) is generally used to confirm positivity and can characterize the immune responses to specific proteins *of Borrelia burgdorferi s.l*. complex. The test kit manufacturers clearly define the interpretation for positive, negative, and equivocal samples.

The European Union Concerted Action on Lyme Borreliosis/EUCALB has conducted a multicenter study for the standardization of the interpretative criteria of immunoblot results in Europe. Although a set of eight bands were identified as significant in each participant laboratory, no single rule was formulated for use across Europe (160). The sensitivity of serological tests for diagnosis of LB is highly heterogeneous, varying with clinical manifestations (161). Average sensitivity estimates of 50% for erythema migrans, 77% for neuroborreliosis, 97% for acrodermatitis chronica atrophicans, and 73% for unspecified LB have been reported (162). Overall, the mean sensitivity of the serologic test was reported in a meta-analysis to be 59.5% (range: 30.6–86.2%) (163). Most European and North American guidelines recommend searching for intrathecal antibody production for the diagnosis of early Lyme neuroborreliosis (156).

In recent years, other commercially available serological tests have been developed for Borrelia detection. Among them, the TickPlex assay is an ELISA-based test, which also contains a new antigen for round bodies/persister forms of Borrelia. This assay has been reported to be useful in different stages of LB and the upgraded test also allows to simultaneously determine IgM and IgG antibodies of several tick-transmitted bacterial and viral pathogens (https://www.arminlabs.com/en/tests/tickplex).

### Direct Detection of Borrelia

Direct detection of *B. burgdorferi sensu lato* can be achieved by culture of the infectious agent, by microscopy, and by the use of molecular methods for the detection of Borrelia nucleic acids. These methods vary in sensitivity and procedure complexity. They can provide evidence for the presence of intact spirochetes or spirochete components, such as DNA or protein, in tick vectors, reservoir hosts, or patients.

#### Culture

Although *in vitro* cultivation of *Borrelia* from clinical samples represents the golden standard for proving an active infection, this method cannot be routinely used for diagnosis as it is time consuming and has low clinical sensitivity (54, 164). *Borrelia burgdorferi sensu lato* culture can be obtained from various tissues and body fluids with variable yield using dedicated media, such as the modified Kelly-Pettenkofer medium (MKP), the Barbour-Stoenner-Kelly II (BSK-II) medium, and the commercially available BSK-H medium (165, 166). *Borrelia* cultivation from clinical samples is mostly successful from skin biopsy when compared to blood and CSF cultures (165, 167).

#### Microscopy

*Borrelia burgdorferi sensu lato* detection by light microscopy is not feasible in clinical practice. The low *Borrelia* load does not allow a direct recognition of the spirochetes in tissue slides for routine diagnostic procedures. However, for specific purposes, the Warthin-Starry's silver stain (168, 169) and more recently the focus floating microscopy (FFM) (170–173), which are light microscopy-based techniques, can be used to detect *Borrelia* in clinical tissues. In addition, Borrelia species were also detected by electron microscopy in human samples from myocardial tissues (174) and crystalline keratopathy (175).

#### PCR

Among molecular methods of detecting *Borrelia*'s nucleic acids, PCR-based methods are the most widely used for confirmation of *Borrelia* infection (167). However, *Borrelia* diagnosis continues to be very difficult, even by PCR (176). PCR sensitivity for *Borrelia* diagnosis is, indeed, highly variable, because of the multiple factors involved in its detectability by PCR. The type of starting material (blood, skin biopsies, cerebrospinal fluid, synovial fluid), the DNA extraction protocols, the possible use of systems for enrichment of microbial DNA, the PCR targets and PCR approach (nested PCR, real time PCR, digital PCR, PCR followed by hybridization, etc.) influence PCR sensitivity (167, 177, 178). The variability in specimens mentioned above and target amplification have also been found in the CE-IVD PCR assays developed for Borrelia detection (177). Low bacterial concentration is the main concern, and a further hypothesis regarding the possibility that during infection *Borrelia* invades the intracellular niche has been suggested (176). Moreover, different non-motile atypical morphologies of *B. burgdorferi* (s.l.) spirochetes have been reported. These include looped or ring-shaped forms, blebs, round bodies, and cell wall deficient forms; spirochete colonies or biofilm aggregates have also been described. The above-mentioned morphologies can impact Borrelia detectability by PCR. Biofilm busters to increase Borrelia load have been suggested for more accurate PCR tests (144). Borrelia PCR from skin biopsy from patients with ECM and ACA usually has a higher rate of positivity, but with large variation among studies (167). However, as the lesions are *per se* pathognomonic of LB, PCR is now only used for research purposes for those lesions. The diagnostic sensitivity of PCR in body fluids is highly variable, depending on the sample type, on the volume of the sample and on the contamination from PCR inhibitors (179). In synovial fluid, PCR for Borrelia detection is more sensitive than in blood and CSF (167). Borrelia targets for PCR must be genetically stable and should enable the detection of all pathogen of Borrelia species. They can be located on the chromosome or on plasmid DNA. The most frequent chromosomal targets that have been reported in clinical studies are flagellin (26, 164, 180–182), 16S rRNA gene (180, 183–185), the gene codifying for the 66 kDa protein (26, 56, 184, 185), while the most used plasmid target is OspA (56, 180, 183, 186–188), which has been also reported to be more stable after degradation of spirochetes (178). At present the major concern in Borrelia diagnosis by PCR is the lack of standardization of the protocols and analyzed targets (167, 177, 178). This heterogeneity in terms of PCR protocols and samples makes it difficult to diagnose LB unequivocally by PCR in settings in which the pre-test probability of LB is very low, including for instance patients suspected of late LB, with negative serology (178).

### Novel Approaches in Borrelia Detection

Because of the limits of serology in detecting the *Borrelia sensu lato* complex in clinical samples, other commercially available tests have been developed. Among them, the T cell response tests, including the lymphocyte transformation test (LTT and MELISA) and the enzyme linked immuno-spot (EliSpot) test have been commercialized. They are based on the detection in patients' blood of Borrelia-specific T-lymphocyte, notably the T helper lymphocytes, which are reported to circulate in the blood in detectable numbers only during an active immune response against Borrelia and to persist in a non-florid infection in lymphoid organs (189).

Alternative tests to the traditional serology and PCR for Borrelia detection have also been proposed. Among them, Luminex-based approaches for Borrelia detection have been reported. This multiplex- high-throughput technique was used for the simultaneous detection of the plasmid contents of different B. burgdorferi strains (10 Ag-Luminex technology) (190), but also to diagnose *Borrelia miyamotoi* in the serum of European patients (191) as well as for the simultaneous detection of 10 insect-borne pathogens, including Borrelia (192). An immuno-PCR (iPCR) assay, which takes advantage of the PCR properties to increase the sensitivity of standard ELISA (193), was also developed and evaluated for the detection of antibodies to the *B. burgdorferi* C6 peptide (194). Other approaches refer to the metabolic profiling for early Lyme disease (195) and the measurement of IFN-γ after incubating blood with *Borrelia* antigens. The latter method was reported to be potentially useful in the laboratory diagnosis of early Lyme disease, even after antibiotic treatment (196).

## Ethics Statement

Written informed consent was obtained as part of the hospital procedures from the individuals and/or minor' legal guardian/next of kin for the publication of any potentially identifiable images included in this article.

## Author Contributions

GT managed the clinical aspect of the review, MR the section dedicated to serology, SB the section related to direct diagnosis go LB. All authors drafted and revised the manuscript.

## Conflict of Interest

The authors declare that the research was conducted in the absence of any commercial or financial relationships that could be construed as a potential conflict of interest.
